# Adult Onset Still's Disease Associated with* Mycoplasma pneumoniae* Infection and Hemophagocytic Lymphohistiocytosis

**DOI:** 10.1155/2016/2071815

**Published:** 2016-10-26

**Authors:** Abhishek Agnihotri, Allison Ruff, Lauren Gotterer, Addie Walker, Amy H. McKenney, Andrei Brateanu

**Affiliations:** ^1^Department of Medicine, Johns Hopkins University School of Medicine, Baltimore, MD, USA; ^2^Department of Internal Medicine, University of Michigan, Ann Arbor, MI, USA; ^3^Department of Neurology, Cleveland Clinic, Cleveland, OH, USA; ^4^Department of Pathology, Cleveland Clinic, Cleveland, OH, USA; ^5^Department of Internal Medicine, Cleveland Clinic, Cleveland, OH, USA

## Abstract

Adult Onset Still's Disease (AOSD) is a systemic inflammatory disorder that can be associated with hemophagocytic lymphohistiocytosis (HLH), a rare but potentially fatal disease of overactive histiocytes and lymphocytes. We present a unique case of AOSD complicated by* Mycoplasma pneumonia* infection and HLH. A 28-year-old female developed joint pains followed by a diffuse, erythematous, pruritic skin rash that quickly spread throughout the body. The patient deteriorated and developed fever, chills, cough, and dyspnea and had to be intubated. She had hypoalbuminemia, elevated liver enzymes, a very high serum ferritin level, positive anti-*Mycoplasma pneumonia* IgG and IgM antibodies, and normal rheumatoid factor and anti-nuclear antibodies. The chest X-ray showed diffuse bilateral infiltrates. Bone marrow biopsy revealed hemophagocytosis. The patient was treated with azithromycin, methylprednisolone, and anakinra and was discharged home on cyclosporine and prednisone. This case highlights that patients can develop features of both AOSD and HLH at the beginning of the disease and early diagnosis and treatment increase the likelihood of recovery.

## 1. Introduction

Adult onset Still's Disease (AOSD) is a systemic inflammatory disorder characterized by prolonged fever, polyarthralgia, and an evanescent rash [1]. The etiology is unknown and infectious agents have been suggested to be triggers in predisposed hosts [2]. Important laboratory features include leukocytosis and hyperferritinemia. The extremely high levels of ferritin encountered in AOSD can also be found in hemophagocytic lymphohistiocytosis (HLH), a rare but potentially fatal disease of overactive histiocytes and lymphocytes [3]. HLH and AOSD are sometimes reported together, suggesting a possible common pathogenic mechanism.

We report a unique case of AOSD complicated by* Mycoplasma pneumoniae* infection and HLH. Informed consent has been obtained from the patient for the publication of this case report.

## 2. Case Report

A 28-year-old female with no past medical history, taking no medications or over-the-counter drugs, developed pain and stiffness in the metacarpophalangeal and proximal interphalangeal joints of both hands, two months after migrating from South Asia to United States. This was immediately followed by the development of a diffuse, erythematous, pruritic skin rash that quickly spread throughout the body. The patient was seen in an outpatient clinic, was diagnosed with an allergic illness, and received oral antihistamines. The symptoms did not improve and the patient deteriorated and developed fever, chills, cough, and dyspnea. She then presented to an outside hospital and was admitted with respiratory failure. After 14 days she was transferred to our hospital for further evaluation and treatment.

**Figure 1:**
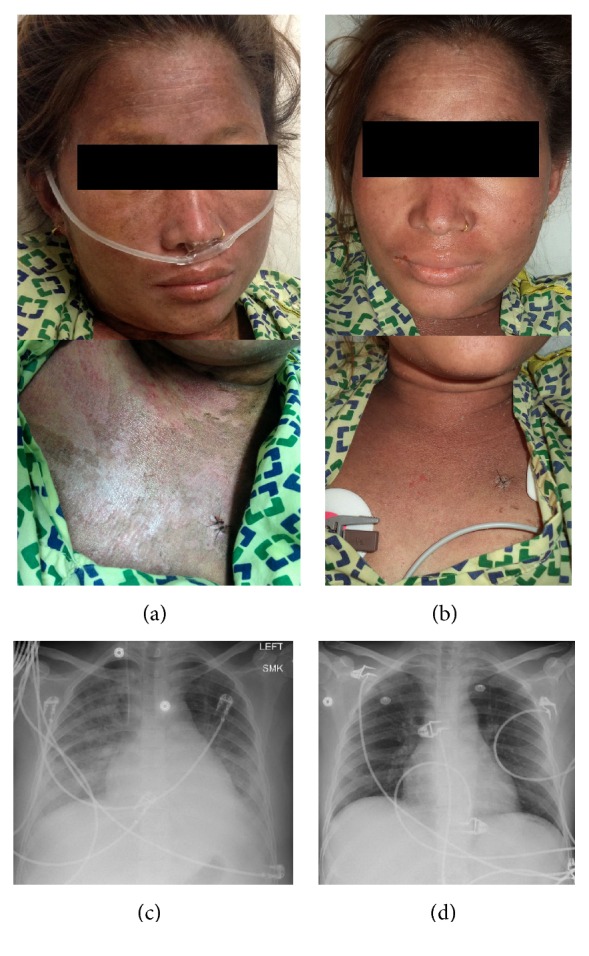
Photograph of the rash and chest X-ray at presentation and discharge. (a) Face and neck upon presentation. (b) Face and neck upon discharge. (c) Chest X-ray at presentation showing bilateral infiltrates. (d) Chest X-ray at discharge showing resolution of infiltrates.

On arrival, vitals were temperature 39.2°C, blood pressure 90/54 mm Hg, heart rate 80/min, respiratory rate 22/min, and pulse oxygen saturation (SpO2) 98% on 2 L nasal cannula. Patient had a diffuse desquamating pruriginous rash (Figure 1), periorbital edema and icterus, and bilateral lung crackles. Laboratory studies revealed anemia (hemoglobin 8.0 g/dL, N: 11.5–15.5 g/dL), hypoalbuminemia (albumin 1.8 g/dL, N: 3.5–5.0 g/dL), hypocalcemia (calcium 6.0 mg/dL, N: 8.5–10.5 mg/dL), and elevated liver enzymes (total bilirubin 2.9 mg/dL, N: 0.0–1.5 mg/dL; alkaline phosphatase 344 U/L, N: 40–150 U/L; ALT 639 U/L, N: 0–45 U/L; AST 1713 U/L, N: 7–40 U/L).* Mycoplasma pneumoniae* IgG and IgM antibodies were increased (1.32, and 1.44, resp., N < 0.91). Tuberculosis (TB) antigen and mitogen response were negative, as was the remainder of the infectious evaluation. The d-dimer levels were >35200 ng/mL, the maximum measurable limit. The serum ferritin level was 167357 ng/mL (N < 150 ng/mL). The rheumatoid factor (RF) and the anti-nuclear (ANA), anti-Sm, anti-ribonucleoprotein, anti-SSA, anti-SSB, anti-centromere, anti-Jo1, anti-ribosomal RNP, anti-chromatin, anti-PL4, anti-neutrophil cytoplasmic, anti-cardiolipin, and anti-Babesia antibodies were all negative.

Chest X-ray showed bilateral infiltrates (Figure 1). Bone marrow biopsy revealed a hypercellular marrow with granulocytic hyperplasia, erythroid hypoplasia with maturation arrest of proerythroblast stage, increased histiocytes, and hemophagocytosis and increased cytotoxic T-lymphocytes. Immunostaining showed increased interstitial CD3 positive T-cells with strong Granzyme B reactivity and dim CD56 expression. Flow cytometry showed increased cytotoxic T-lymphocytes. Skin biopsy of the evanescent rash revealed hyperkeratosis and focal parakeratosis of the stratum corneum. Numerous apoptotic keratinocytes were appreciated in clusters and singly within the stratum corneum, suggestive of Still's Disease [4, 5].

The patient fulfilled all the four major and three of the four minor criteria as described by Yamaguchi et al. [6] and was diagnosed with AOSD. Similarly, she fulfilled six out of eight criteria for the diagnosis of HLH [7]. The nonclassical pruritic rash [4] was considered to be part of the atypical presentation of AOSD and not an allergic reaction, considering that patient had no medication history prior to admission.

**Figure 2:**
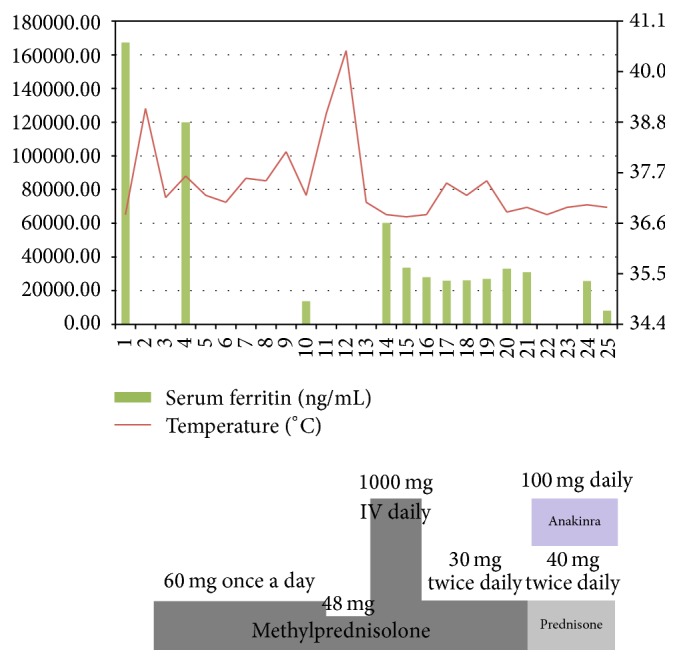
Serum ferritin levels and temperature charting along with the immunosuppression.

The patient began treatment with oral azithromycin 500 mg daily and methylprednisolone 60 mg daily. The rash improved and fever subsided. When tapering the dose of methylprednisolone to 48 mg daily was attempted, the patient developed a high-grade fever to 40.5°C. She was transferred to the intensive care unit and received intravenous methylprednisolone 1000 mg for 3 days followed by anakinra 100 mg subcutaneous daily along with prednisone 40 mg twice daily (Figure 2). After 6 more days she improved and was discharged home on cyclosporine 50 mg twice daily and prednisone 30 mg in the morning and 20 mg in the evening with a plan for a slow weekly taper.

## 3. Discussion

We describe a case of AOSD complicated by* Mycoplasma pneumoniae* infection and HLH. HLH has been frequently described as a complication of AOSD [8] and is usually referred to as macrophage activation syndrome (MAS) when associated with AOSD or its equivalent in children, systemic juvenile idiopathic arthritis [8]. It is unclear if HLH is one end of the spectrum of AOSD given that they share many clinical and laboratory features such as fever, hepatosplenomegaly, rash, hyperferritinemia, coagulopathy, and elevated liver enzymes [6, 7, 9]. The Yamaguchi criteria for the diagnosis of AOSD include fever, arthralgia, typical rash, and leukocytosis as major and sore throat, lymphadenopathy, hepatomegaly or splenomegaly, liver dysfunction, and the absence of rheumatoid factor and anti-nuclear antibody as minor criteria [6]. Requiring 5 or more criteria and including 2 or more major ones yield a 96.2% sensitivity and 92.1% specificity [6]. However, an exclusion process is needed for an accurate classification, since this disease is relatively rare. On the other hand, the diagnosis of HLH may be established by the fulfillment of five out of the following eight criteria: fever, splenomegaly, cytopenias, hypertriglyceridemia, hemophagocytosis, low or absent NK cell activity, hyperferritinemia, and soluble CD25 (IL-2 receptor) [7]. Since HLH is a potentially fatal condition, early diagnosis and treatment are key.

It is possible that the* Mycoplasma pneumoniae* infection may have precipitated HLH in the background of AOSD. However, the association between* M. pneumonia* infection and HLH has primarily been reported in children [10, 11]. It usually has a good prognosis and cases resulting in patients' death were those where immunosuppression was delayed secondary to a delayed diagnosis [12, 13].

Coffernils et al. suggested that markedly elevated serum ferritin levels should raise the suspicion of HLH and warrant a bone marrow study [14]. However, demonstration of hemophagocytosis in bone marrow is only one of the criteria in the diagnosis of HLH and bone marrow histology is not always necessary in diagnosing HLH in AOSD patients, considering patients' inconvenience and benefit [7]. Moreover, hemophagocytosis might not be present in the initial stages of the acquired forms of HLH due to autoimmune or inflammatory diseases. Thus, the detection of macrophage hemophagocytosis on bone marrow biopsy specimens is not required for the diagnosis of MAS [15]. Also, in order not to delay the diagnosis, the new classification criteria include only laboratory (high ferritin, triglycerides and aspartate transaminase, and low platelet count and fibrinogen level) and no clinical variables, with the exception of fever [16]. Although designed for use in clinical research, criteria can be applied in individual patients.

Treatment of AOSD is empirical, based on managing symptoms using nonsteroidal anti-inflammatory drugs (NSAIDs), steroids, and antirheumatic agents [2]. NSAID monotherapy is effective in only 7–15% of the cases and most patients are also treated with steroids, with a positive response ranging from 76 to 95% [17]. The HLH treatment protocol developed by the International Histiocyte Society recommends treatment with steroids, etoposide, and cyclosporin A [7]. Biologic agents can be used in refractory cases that do not respond to corticosteroids [18, 19], although some of these drugs have been reported to induce HLH in AOSD patients [20, 21]. Tumor necrosis factors (TNF) inhibitors seem to be useful in the chronic polyarticular form of the disease [18, 22]. The recombinant antagonist of the IL-1 receptor, anakinra, has been used successfully in the refractory AOSD with systemic symptoms [18, 23]. Furthermore, anakinra is not associated with an increased risk of adverse events [24] and has been successfully used in patients with HLH complicating AOSD [18].

Although both AOSD and HLH have similar treatment approaches, it should be noted that while AOSD is usually a more benign disease that can become chronic, HLH is a deadly disease warranting early diagnosis and prompt treatment. In our case, the use of anakinra was delayed due to the risk of reactivation of latent tuberculosis, owing to the patient's South Asian origin. This weighed against its use but when the patient deteriorated, the benefits were determined to outweigh the potential risk. This highlights an important aspect of infection-induced HLH, where the use of immunosuppression has to be balanced against the possibility of worsening the primary infectious process. It should be noted that early tapering of steroids should be avoided, as there may be a sudden deterioration of the patient's condition.

In conclusion, HLH may concomitantly occur in addition to the first features of AOSD, which might alter the typical clinical features of the disease. Early diagnosis and treatment with high dose steroids and in refractory cases with biologic agents are critical in achieving a positive outcome.
